# Weibel-Palade bodies: function and role in thrombotic thrombocytopenic purpura and in diarrhea phase of STEC-hemolytic uremic syndrome

**DOI:** 10.1007/s00467-024-06440-3

**Published:** 2024-07-05

**Authors:** Leo Monnens

**Affiliations:** Department of Physiology, Radboud University Centre, Nijmegen, the Netherlands

**Keywords:** STEC-HUS, Weibel-Palade bodies, Von Willebrand factor, P-selectin, TTP, Endothelial cells

## Abstract

**Abstract:**

Vascular endothelial cells are equipped with numerous specialized granules called Weibel-Palade bodies (WPBs). They contain a cocktail of proteins that can be rapidly secreted (3–5 min) into the vascular lumen after an appropriate stimulus such as thrombin. These proteins are ready without synthesis. Von Willebrand factor (VWF) and P-selectin are the main constituents of WPBs. Upon stimulation, release of ultralarge VWF multimers occurs and assembles into VWF strings on the apical side of endothelium. The VWF A1 domain becomes exposed in a shear-dependent manner recruiting and activating platelets. VWF is able to recruit leukocytes via direct leukocyte binding or via the activated platelets promoting NETosis. Ultralarge VWF strings are ultimately cleaved into smaller pieces by the protease ADAMTS-13 preventing excessive platelet adhesion. Under carefully performed flowing conditions and adequate dose of Shiga toxins, the toxin induces the release of ultralarge VWF multimers from cultured endothelial cells. This basic information allows insight into the pathogenesis of thrombotic thrombocytopenic purpura (TTP) and of STEC-HUS in the diarrhea phase. In TTP, ADAMTS-13 activity is deficient and systemic aggregation of platelets will occur after a second trigger. In STEC-HUS, stimulated release of WPB components in the diarrhea phase of the disease can be presumed to be the first hit in the damage of Gb3 positive endothelial cells.

**Graphical abstract:**

**Figure:**
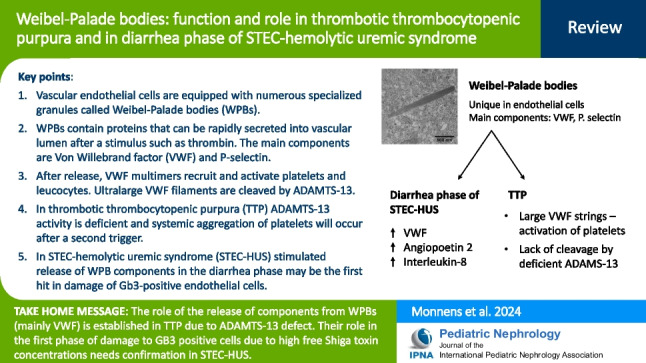
A higher resolution version of the Graphical abstract is available as Supplementary information

**Supplementary Information:**

The online version contains supplementary material available at 10.1007/s00467-024-06440-3.

## Introduction

Vascular endothelial cells line the luminal surface of the vasculature. Endothelial cells contain unique storage organelles, so called Weibel-Palade bodies (WPBs). In these WPBs two main proteins are stored, namely von Willebrand factor (VWF) and P-selectin.

**Fig. 1:**
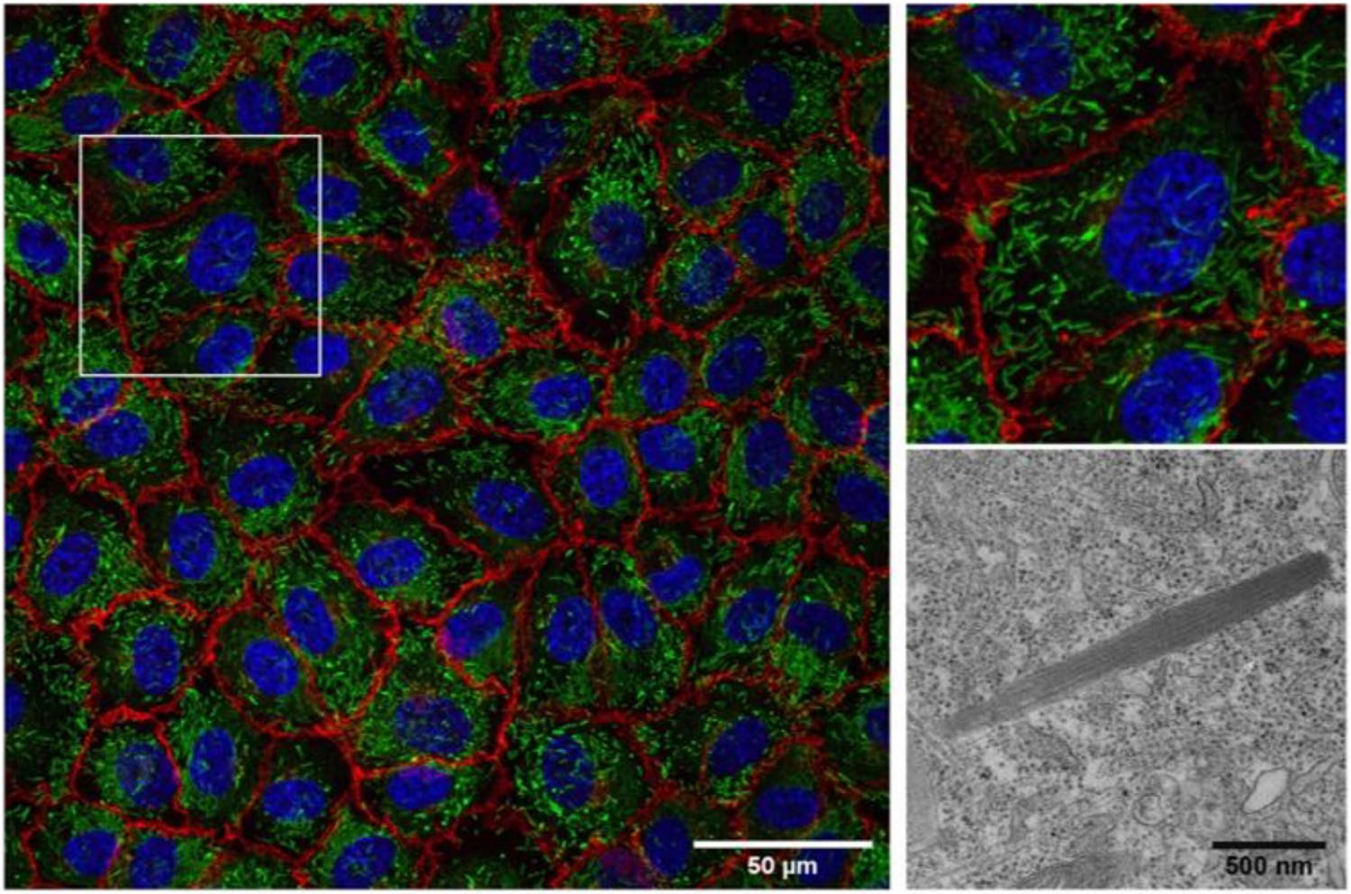
von Willebrand factor in endothelial cells. Fluorescence microscopy image of cultured human endothelial monolayer (HUVEC) immunostained for VWF (green) and nuclei (blue). Electron microscopy of Weibel-Palade bodies. Courtesy of Marije Kat

WPBs are rod-shaped, elongated structures containing tubules formed by polymerized VWF and aligned parallel to the longitudinal axis of WPBs [1] (Fig. 1). Although the major component within WPBs is VWF, these vesicles also contain a number or other bioactive molecules. Expression of VWF drives the formation of WPBs [2].

Data on the list of proteins residing in WPBs and insight into the dynamics and regulation of exocytosis will be presented with emphasis on their role in the pathogenesis of TTP and hemolytic uremic syndrome secondary to Shiga toxin-producing *Escherichia coli* (STEC-HUS) in the diarrhea phase.

## Weibel-Palade bodies—components

Multiple components are co-stored with VWF in WPBs. VWF will be covered in a later section.

### P-selectin

P-selectin is a single-chain glycoprotein with a molecular weight of 140 kDa and is stored in the α granules of platelets and WPBs, and rapidly exposed on the surface of these cells upon stimulation. Transmembrane P-selectin is present in dimers or oligomers whereas soluble P-selectin in plasma, resulting from proteolytic cleavage of transmembrane P-selectin, is in the monomeric form and unable to interact with functional ligands [3].

P-selectin binds to leucocyte P-selectin glycoprotein ligand-1 (PSGL1) so that the leucocytes become loosely attached rolling along the vessel wall, leading to firm adhesion and transmigration [4].

Following its expression on the cell membrane P-selectin is rapidly internalized, transported to early and late endosomes from where it can be recycled independent of de novo protein synthesis. The recycled P-selectin retains its adherence function [5]. In addition, P-selectin increases via PSGL1, the expression of tissue factor on monocytes [6].

#### CD63

After exocytosis of WPBs, the membrane glycoprotein CD63 is transported to the cellular surface where it forms clusters with P-selectin. CD63 is an essential cofactor for P-selectin function [7, 8]. CD63 is not exclusively present within WPBs but also localizes to lysosomes and late endosomes [9]. P-selectin is already included during WPB formation due to direct binding to VWF. CD63 is trafficked from endolysosomes via two-pore channel 2 (TPC2) [10]. TPC2 governs leukocyte capture on endothelium.

### Endothelin

Endothelin is synthesized in human endothelial cells [11]. The constitutive pathway via small secretory vesicles maintains vascular tone [12]. Via binding to ET_A_ and ET_B_ receptors on vascular smooth muscle cells, an initial transient vasodilatation followed by marked sustained vasoconstriction is induced. In the regulatory pathway ET-1 is released from WPBs situated in the endothelial cells. ET-1 is generated by endothelin-converting enzyme from big ET-1. ET-1, the major endothelial isoform, is secreted on the abluminal surface, remarkably different from the exocytosis of VWF. Plasma concentrations of ET-1 do not accurately reflect ET-1 production. Apparently a small amount is passing the luminal side. The half-life of ET-1 in the healthy circulation is about 1 min [13]. The effect of ET-1 on the glomerulus has been extensively studied and will be further discussed, omitting the effect on other structures such as tubular cells. Glomerular endothelial cells are probably the principal but not unique source of ET-1 within the glomerulus. ET-1 secreted abluminally modulates podocyte and mesangial structure and function. ET-1 induces cytoskeletal (actin) remodeling in podocytes and loss of slit-diaphragm proteins such as nephrin. It increases glomerular permeability reflected in albumin excretion [14]. ET-1 expression in cultured podocytes by Shiga toxin mediates cytoskeleton rearrangements in the podocytes [15]. Podocyte activation via endothelin results in loss of endothelial surface layer (glycocalyx) [16]. In transgenic mice with GB3 expression exclusively in their podocytes, Shiga toxin induced a loss of endothelial glycocalyx with reduction of complement factor H binding [17]. This reduction has consequences for complement activation [18] and can have a role for the preferred damage of the glomeruli in STEC-HUS. Endothelial-targeted treatment has a beneficial effect in a wide range of kidney disorders [19, 20].

### Calcitonin gene-related peptide (CGRP)

CGRP is the most potent microvascular vasodilator. CGRP is released from perivascular nerves. The major effects are exerted locally in the vessel wall close to its site of synthesis acting on the arterioles, causing vasodilatation and influencing the cardiovascular system. CGRP mediates this response by directly activating its receptors on the vascular smooth muscle and on endothelial cells to enhance NO production [21, 22]. Despite its potent action on the vasculature, CGRP does not play a pivotal role in the normal physiological regulation of blood pressure. Endothelin-converting enzyme (present in WPBs) mediates degradation of CGRP [23]. Is CGRP present in WPBs mitigating the effect of endothelin released from WPBs?

### Osteoprotegerin (OPG)

OPG is an effective inhibitor of osteoclast differentiation and activation. Produced by osteoblasts it suppresses osteoclast formation impeding the binding of RANKL with RANK.

Endothelial cells and vascular smooth muscle cells have been demonstrated to produce OPG [24, 25]. Not all exposed platelet-binding sites on VWF secreted by endothelial cells are occupied by platelets. OPG interacts with the VWF A1 domain and is responsible for the reduced binding to platelets [26].

### Angiopoetin-2 (Ang-2)

Ang-1 is constitutively expressed by many different cell types in contrast to the expression of Ang-2, which is almost exclusively expressed in endothelial cells. Ang-1 mediates Tie2 activation and is required to maintain the quiescent resting state of the endothelium. Ang-2 functions as an antagonist ligand for Tie2. It results in destabilization, rendering endothelium responsive to stimulation by inflammatory cytokines such as TNF, interleukin-1, and angiogenic cytokines (VEGF) [27] and induces permeability of endothelium.

VWF binds to Ang-2 via the VWF A1 domain, persisting after secretion [28]. Is VWF mediating the storage of Ang-2? The intracellular Ang-2 stores rapidly recover upon release. It is detectable 6 h after complete release and recovered within 16 h [29]. A repeated stimulation is possible as is also shown for P-selectin.

### Ribonuclease 1 (RNase 1)

Extracellular RNAs exist in the extracellular space and are not inert molecules. They induce hyperpermeability of endothelium, and enhance adhesion and transmigration of leukocytes. Furthermore, they induce the release of cytokines from monocytes/macrophages, activate the inflammasome, and potentiate blood coagulation [30, 31]. Human ribonuclease A degrades RNAs in the extracellular space. Insight into the biological function of RNase 1 is sparse but it has a protective effect [32]. Only ribonucleases that evade RNase may be cytotoxic.

The role of a 1,3-fucosyltransferase VI (Fuct-VI) is unclear [33].

### Interleukin-8, MCP-1

Resting endothelial cells do not synthesize interleukin-8 (IL-8) in significant amounts [34]. De novo synthesis of IL-8 and monocyte chemoattractant protein (MCP-1) require exposure to cytokines such as interleukin-1β, IL-4, and TNF for a prolonged period (24 h) [35].

IL-8, IL-6, MCP, tissue activator (tPA), and growth-regulated oncogen-a were found to reside in both WPBs and small punctate vesicles in human endothelial cells. It is suggested that they are missorted to WPBs. Low amounts of the de novo synthesis of IL-8 and tPA are sorted into WBPs. The storage of these proinflammatory mediators equips endothelial cells with a rapidly recruitable reservoir.

The chemokines IL-8 and MCP regulate leucocytes and monocyte movement by adhesion and extravasation including activation [36, 37].

## Proteomic analysis

A proteomic screen identified novel components of endothelial cell-specific WBPs, such as IGFBP7 with a suggested role in angiogenesis [38].

Taking into account the different components of WPBs, their release will provoke fire. Can this fire be extinguished (partially) by interfering with the synthesis of VWF and blockage of P-selectin action?

## Stimulation of release

A large, still increasing variety of diverse agonists can trigger exocytosis of WPBs [39]. Ca^2+^-mediated and cAMP-mediated secretagogues converge at effector pathways that control anchoring tethering vesicle fusion and actin contractility [2]. Release of WPBs has been induced by fibrin [40], thrombin, endothelin [41], C5a [42], C5b-9 [43], inflammatory cytokines [44], activated platelets [45], and heme. A continuous stimulation of release of WPBs probably occurs in TTP and STEC-HUS. Platelets express high-mobility group box 1 protein (HMGB1), and upon platelet activation, HMGB1 is exported to the cell membrane and released [46]. It induces monocyte tissue factor expression initiating coagulation, platelet activation, and NETosis [47]. In a murine model of typical HUS with increased plasma HMBGB1, administration of anti-HMBGB1 promoted amelioration of tissue damage. The Shiga toxins have an enzymatically active A moiety and non-toxic B moiety. The B moiety consists of five identical B subunits forming a pentameric ring. Each B subunit harbors three distinctive binding sites that interact with the trisaccharide moiety of the glycosphingolipid Gb3 [48]. A seminal study Nolasco et al. [49] demonstrated that Shiga toxin under flowing conditions stimulated the secretion of long VWF multimeric strings in viable human umbilical vein endothelial cells (HUVEC) and human glomerular microvascular endothelial cells (GMVEC) within 10 min. Perfused human platelets immediately adhered to these strings. In addition, Shiga toxin impairs ADAMTS-13 cleavage by binding to the A2 domain [50]. This delayed VWF cleavage may contribute to renal thrombotic microangiopathy in STEC-HUS. The B subunits of Stx1 and Stx2 in a perfusion assay stimulated the secretion of ultralarge VWF from HUVEC within 5 min [51].

More details about the exocytosis will be covered in the next section about VWF.

## Role of Von Willebrand factor (VWF)

### Biosynthesis, secretion, and clearance

Excellent reviews covering biosynthesis, secretion, and clearance can be found in more detail elsewhere [52–56]. To understand the role of VWF, however, it is important to have some background in this respect. The contribution of VWF from α granules of platelets requires platelet activation and is limited. In endothelium, in non-stimulated condition, VWF is replaced by approximately 50% after 24 h. Briefly, following synthesis of ProVWF monomers, dimers are formed in the endoplasmic reticulum and transported in vesicles to the Golgi. Once arrived at the Golgi, the propeptide is cleaved, and VWF dimers multimerize and form quanta that arrive in the transGolgi network to be packaged into WPBs. A complex pathway is followed along intraendothelial storage and trafficking combined with basal and regulated secretion.

VWF secretion occurs via three pathways: constitutive secretion of low molecular VWF primarily released at the basolateral side of the endothelium and basal and regulated secretion of high molecular VWF to the apical surface. From the large number of WPBs undergoing exocytosis upon stimulated release, ultralarge WPB multimers assemble into VWF strings on the apical side of the endothelium [57]. The large multimers have increased platelet adhesive capacity compared with the low molecular VWF [58].

Three modes of exocytosis can be distinguished. WPBs can undergo full fusion, resulting in complete release of VWF and other WPB compounds. Incomplete fusion, so called lingering kiss, occurs via small fusion pore. It allows only release of small proteins, such as IL-8, but not of VWF and P-selectin [59]. About 25% of WPBs may undergo lingering kiss fusion after stimulation. In a third mode, multiple WPBs aggregate and fuse to the membrane. The fusion of WPBs with the membrane is facilitated by SNARE complex formed by proteins which are present on vesicles (v-SNAREs) and target membranes (t-SNAREs) [2, 55]. Large WPBs undergoing full fusion exploit the contractile properties of actomyosin rings to forcibly release high molecular VWF not affecting the release of P-selectin and other cargo [55, 60].

Following release into the circulation, the half-life of VWF has a large variation ranging from 4.2 to 26 h. Individuals with blood group non-O display a longer half-life than individuals with blood group O due to different glycosylation patterns. Receptor-mediated endocytosis mainly by macrophages has an important role in the clearance of VWF [52, 61].

**Fig. 2:**
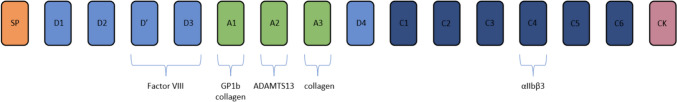
Domain structure of von Willebrand factor (VWF). SP signal peptide, D1–D2 propeptide; domains potentially involved in interactions relevant for inflammatory processes. GP1b and αIIbβ3 are binding sites for platelets

VWF circulates in plasma in a globular form not interacting with platelets. After vascular damage, VWF binds to collagen and uncoils in adhesive strings. This adaptation occurs also after exocytosis of WPBs at the luminal side of endothelium. The endothelial glycocalyx, via binding to heparan sulfate, anchors VWF to the vascular endothelium. VWF is essential for the capture of platelets via two receptors (Fig. 2), GP1b-IX-V and αIIbβ3, and requires flowing blood. A1 domain of VWF becomes exposed under shear stress. In static conditions no binding between VWF and the two receptors is observed [62]. The engagement of αIIβb3 occurs after initial platelet adhesion mediated by GP1b-IX-V/VWF interaction. Platelet activation is induced [63]. Activated platelets release alpha granules containing thrombospondin-1 (TSP-1). TSP-1 competes with the A2 binding site of ADAMTS-13 inhibiting VWF multimer cleavage [64]. Activated platelets expose phosphatidylserine on their membrane allowing the coagulation factor binding properties such as the prothrombinase complex. Activated platelets trigger all three pathways of complement cascade [63, 65]. Circulating VWF, in addition, acts as a chaperone for factor VIII to protect this coagulation factor from proteolytic degradation.

Outside hemostasis, VWF may play an important role in inflammatory response. Following blood vessel injury VWF escapes from plasma to subendothelium, where it comes into contact with tissue-resident macrophages. It will trigger these macrophages to adopt an M1 phenotype with consequent secretion of proinflammatory cytokines and chemokines [66].

## VWF and NETosis

VWF is able to recruit leukocytes either via direct leukocyte binding or by recruiting platelets, which in turn will attract leukocytes. VWF interacts with two distinct leucocyte receptors: PSGL-1 and various β2-integrins [67]. Activated platelets promote NET formation (neutrophil extracellular traps) via soluble factors and direct platelet–neutrophil interaction. In NET formation, neutrophils externalize their decondensed chromatin together with granule proteins (elastase, myeloperoxidase, and cathepsin) with a probable role for chromain decondensation [67, 68]. Individual NET components promote thrombin generation as illustrated by Schulz and Massberg [69]. NETs could also activate the alternative complement pathway [70]. Neutrophil activation in STEC-HUS (via elastase) mediates endothelial injury [71].

## Limiting conditions for endothelial damage in culture

Cultured endothelial cells are frequently used to elucidate the pathogenesis of glomerular endothelial disorders such as STEC-HUS. Experimental approaches are needed to better mimic the in situ situation [72]:

- Study in a flowing system is needed. In a static system the interaction of VWF with platelets is impaired and ADAMTS-13 cleaving activity is lacking.
- There is a lack of interaction with other cell types such as the podocyte.
- Evaluation secondary to a previous interacting factor is not correct.
- Data obtained in HUVEC cannot be extrapolated to GMEC [73, 74]. Substantial phenotype heterogeneity exists in different cultured endothelial cells in expression of VWF, including the response to shear stress [75, 76].

The method applied by Nolasco (Sadler) approaches the clinical situation using HUVEC in a flowing system with adequate dose of STx.

In two out of four cultured glomerular microvascular endothelial cells (GMVECs), using the method of Nolasco, a decrease of intracellular VWF was observed. No effect was observed in static conditions of HUVEC and GMVEC [99].

## Thrombotic thrombocytopenic purpura (TTP)

TTP is characterized by systemic aggregation of platelets within the vasculature (generally arteries and arterioles) causing microvascular thrombosis, hemolytic anemia, and thrombocytopenia. The systemic aggregation is due to lack of VWF cleaving protease ADAMTS-13. This protease is present in plasma and is also synthesized in endothelial cells. Under condition of shear stress, released VWF multimers unfold and assemble into string-like structures and bind platelets. Unfolded VWF multimers expose the VWF A2 domain, which now can be cleaved by ADAMTS-13. No effect is obtained in static conditions.

Even a complete deficiency of ADAMTS-13 is not sufficient to cause TTP [77]. A second hit is required. Infections have been recognized as triggers, although the mechanism is still not completely clear [78, 79]. The majority of patients suffering from acquired TTP develop antibodies that bind and neutralize the proteolytic activity of ADAMTS-13 and enhance its clearance [80]. The mainstay of treatment is ADAMTS-13 replacement with plasma exchange and immunosuppression [81].

Caplacizumab is a bivalent humanized immunoglobulin fragment. It binds to the A1 region of VWF and prevents platelet binding to receptor GPIb-IX-V, thus preventing the formation of platelet-rich thrombi. It has a definite role in the current guideline of management [81, 82]. Targeted ADAMTS-13 replacement therapy could help to manage acute episodes of TTP [83].

Congenital TTP is a rare disease due to severe deficiency of ADAMTS-13 caused by mutations. The patients are dependent on regular fresh plasma infusions. Recombinant ADAMTS-13 injections are an effective prophylactic therapeutic approach [84].

## STEC-HUS—diarrhea phase

**Fig. 3:**
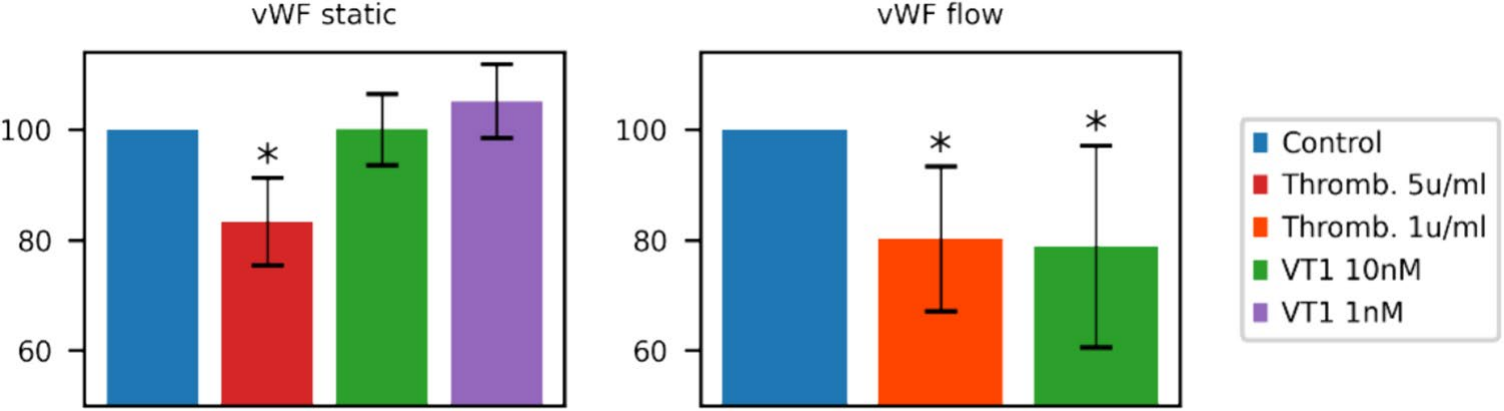
Cell lysate in static condition (left side) and in flow condition (right side) after incubation with thrombin or ST × 1. A star (*) indicates significant values

Endothelial damage occurring during the initial diarrhea phase of STEC-HUS is the first phase in the pathogenesis of this disorder. In a pivotal study, Chandler et al. [85] showed that thrombin generation and inhibition of fibrinolysis preceded renal injury. One of their explanations was the probability that circulating Shiga toxins directly injured renal cells but that the renal manifestations of this injury appeared later. The extremely high concentrations of Shiga toxin measured in the diarrhea phase of family members sustain this concept [86]. As the necessary requirements for safe collection and preservation of plasma samples were unknown, these samples were collected in ice, frozen at − 80 °C, and transported on ice. With a serum half-life of STx2 in mice of 3–3.9 min, some concentrations will remain undetected. As shown by Nolasco and Sadler [49, 51], an immediate effect of Shiga toxin (after 3–5 min) on the release of WPBs (releasing VWF) was observed in a flowing system of cultured endothelial cells (see also Fig. 3). No effect was observed after antibody application to Gb3 receptors. Angiopoetin2/angiopoetin1 ratio was increased in the preclinical phase [87]. VWF decreased size has been observed in the diarrhea phase and after onset of HUS [88]. Plasma P-selectin increased in TTP and after onset of STEC-HUS was not measured. Important data were provided by Yamamoto et al.—IFN-γ, TNF-α, IL-1β, IL-4, IL-6, IL-8, and IL-10 were measured in serum in the diarrhea phase. Only IL-6 and IL-8 were increased, both components of WPBs [89]. This increase in serum IL-8 was confirmed by Westerholt et al. [90]. Contribution of WPB components, stimulated by high Shiga toxin concentration, can be considered a first hit in the damage of Gb3 positive endothelial cells. In addition, serum thrombomodulin concentration, reflecting endothelial damage, was decreased [89, 91]. It is intriguing that in severe Covid-19 infection, the WPB components VWF, angiopoietin-2, and osteoprotegerin were increased in plasma [92]. Shiga toxin, via Gb3 receptor, binds to activated platelets [93], to monocytes [94], red blood cells, and doubtfully to leucocytes [95]. A transport via these cells to Gb3-positive endothelial cells is possible. An innovative concept for transfer of Shiga toxin is revealed by Stahl et al. [96]. The binding of toxin to platelets, monocytes, and red blood cells resulted in the release of extracellular vesicles. These microvesicles can be taken up not only by Gb3-positive but also by Gb3-negative cells. The recipient cell must express endogenous Gb3 for the cell to be susceptible to the toxin [97]. When incubating with glomerular endothelial cells in vitro, Stx2-containing microvesicles bound to the cells and were demonstrated within the cell after 3 h [96]. In vitro studies on cultured glomerular endothelial cells affected cell viability and inhibited protein synthesis. The effect of Stx2 microvesicles, however, was not tested in a flowing system and not taking into account a previous first hit. This first hit will influence the endothelial structure and the availability of Gb3 receptor. Is the first hit sufficient to induce severe endothelial damage, or is subsequent addition of Shiga toxin required? This question was already raised by Tarr et al. [98]. It is striking, as is shown in Fig. 3, that after a high dose of Shiga toxin in a flowing system as applied by Nolasco, the majority of VWF still remains after 15 min, as is visually illustrated by fluorescence microscopy images [99]. A repeated stimulation is possible and the WPB content can be restored. Repeated doses of DDAVP on consecutive days had approximately 30% less response than on the first day [100].

It is assumed that mechanical damage to red blood cells as a result of the formation of microthrombi induces hemolysis. In physiological conditions, cell-free hemoglobin and heme are promptly scavenged by haptoglobin and hemopexin. In hemolytic diseases, the detoxification systems are overwhelmed. In STEC-HUS heme concentration in plasma is increased, supporting a role as a secondary hit. Hemolysis is not present preceding STEC-HUS [85]. Toxicity pathways driven by hemolysis are excellently reviewed [101, 102]. Striking is the inhibition of ADAMTS-13 activity [103], degradation of heparan sulfate of the endothelial glycocalyx inducing local complement activation [104] and promoting rapid exocytosis of WPBs with membrane expression of P-selectin [105].

## Conclusion

The role of the release of components from the WPBs (mainly VWF) is established in TTP due to ADAMTS-13 defect. Their role in the first phase of damage to GB3-positive cells due to high free Shiga toxin concentrations needs confirmation. Endeavors to provide more insight will indicate an effective way of prevention of STEC-HUS.

## Supplementary Information

Below is the link to the electronic supplementary material.

Graphical abstract (PPTX 1042 KB)
