# Supplementary material for: Weibel-Palade bodies: function and role in thrombotic thrombocytopenic purpura and in diarrhea phase of STEC-hemolytic uremic syndrome
Source: Pediatr Nephrol. 2024 Jul 5;40(1):5–13. doi: 10.1007/s00467-024-06440-3 (PMC11584422; doi:10.1007/s00467-024-06440-3)
Supplement: Supplementary file 1 — Graphical abstract (PPTX 1042 KB) [file 467_2024_6440_MOESM1_ESM.pptx]

## Slide 1
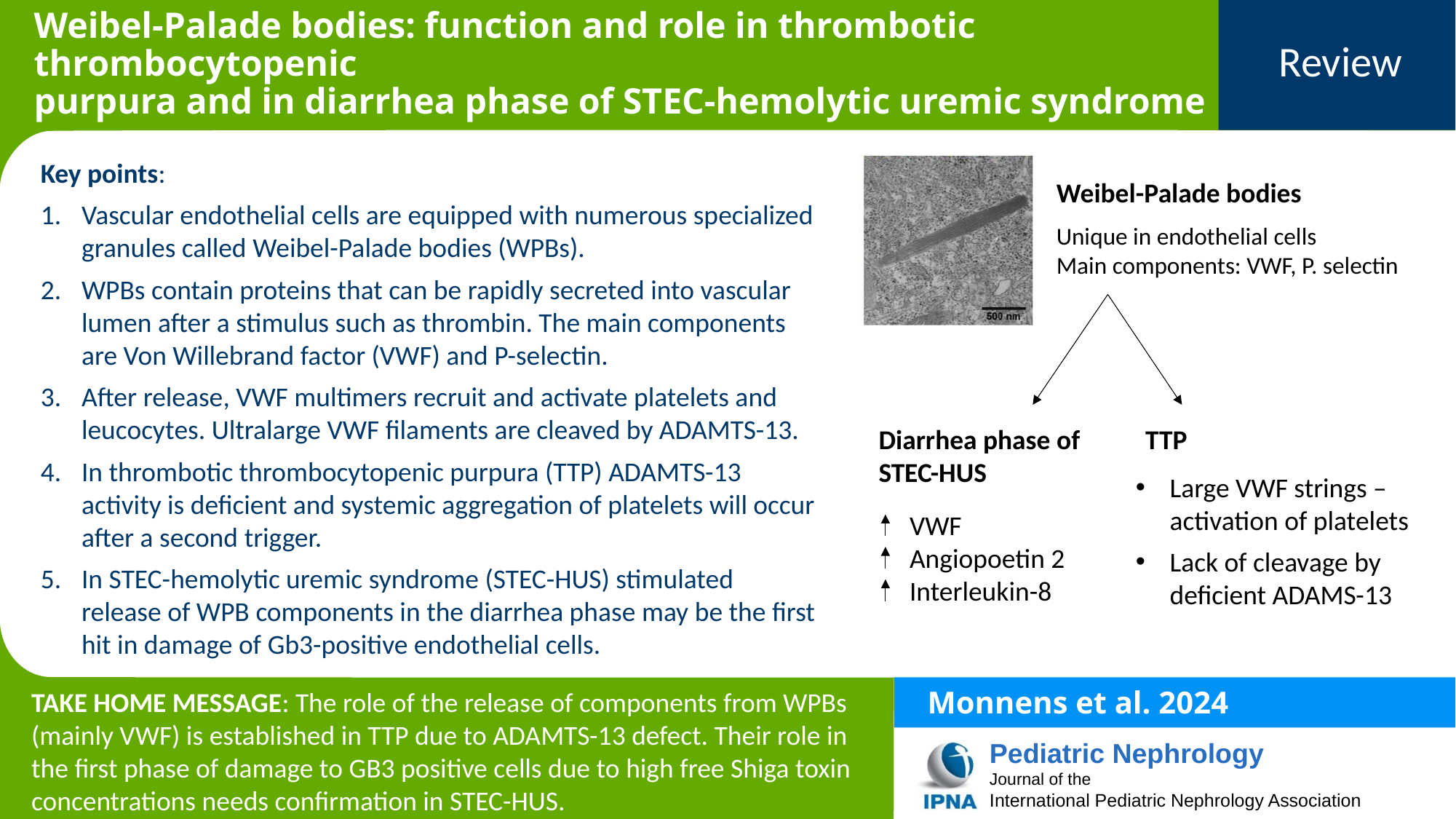

Weibel-Palade bodies: function and role in thrombotic thrombocytopenic
purpura and in diarrhea phase of STEC-hemolytic uremic syndrome
Key points:
Vascular endothelial cells are equipped with numerous specialized granules called Weibel-Palade bodies (WPBs).
WPBs contain proteins that can be rapidly secreted into vascular lumen after a stimulus such as thrombin. The main components are Von Willebrand factor (VWF) and P-selectin.
After release, VWF multimers recruit and activate platelets and leucocytes. Ultralarge VWF filaments are cleaved by ADAMTS-13.
In thrombotic thrombocytopenic purpura (TTP) ADAMTS-13 activity is deficient and systemic aggregation of platelets will occur after a second trigger.
In STEC-hemolytic uremic syndrome (STEC-HUS) stimulated release of WPB components in the diarrhea phase may be the first hit in damage of Gb3-positive endothelial cells.
Weibel-Palade bodies
Unique in endothelial cells
Main components: VWF, P. selectin
Diarrhea phase of STEC-HUS
TTP
Large VWF strings – activation of platelets
Lack of cleavage by deficient ADAMS-13
VWF
Angiopoetin 2
Interleukin-8
Monnens et al. 2024
TAKE HOME MESSAGE: The role of the release of components from WPBs (mainly VWF) is established in TTP due to ADAMTS-13 defect. Their role in the first phase of damage to GB3 positive cells due to high free Shiga toxin concentrations needs confirmation in STEC-HUS.
